# Modulating embryonic signaling pathways paves the way for regeneration in wound healing

**DOI:** 10.3389/fphys.2024.1367425

**Published:** 2024-02-16

**Authors:** Sophie Frech, Beate M. Lichtenberger

**Affiliations:** Department of Dermatology, Medical University of Vienna, Vienna, Austria

**Keywords:** skin regeneration, fibroblast, hair follicle, WIHN, embryonic signaling pathways

## Abstract

Epithelial tissues, including the skin, are highly proliferative tissues with the capability to constant renewal and regeneration, a feature that is essential for survival as the skin forms a protective barrier against external insults and water loss. In adult mammalian skin, every injury will lead to a scar. The scar tissue that is produced to seal the wound efficiently is usually rigid and lacks elasticity and the skin’s original resilience to external impacts, but also secondary appendages such as hair follicles and sebaceous glands. While it was long thought that hair follicles develop solely during embryogenesis, it is becoming increasingly clear that hair follicles can also regenerate within a wound. The ability of the skin to induce hair neogenesis following injury however declines with age. As fetal and neonatal skin have the remarkable capacity to heal without scarring, the recapitulation of a neonatal state has been a primary target of recent regenerative research. In this review we highlight how modulating dermal signaling or the abundance of specific fibroblast subsets could be utilized to induce *de novo* hair follicles within the wound bed, and thus to shift wound repair with a scar to scarless regeneration.

## 1 Introduction

Mammalian skin comprising the epidermis with its appendages - hair follicles (HFs), sebaceous and sweat glands - and the dermis, has evolved the remarkable ability to self-renew and repair itself upon injury (Fuchs, 2007; Watt and Fujiwara, 2011). However, every injury will lead to a scar. Amazingly, embryonic skin has the exceptional capability for scarless wound healing (Leavitt et al., 2016). Similarly, the formation of HFs from the developing epidermis, which depends on reciprocal signaling between cells of the epidermis and a condensate of mesenchymal cells of the underlying dermis which subsequently forms the dermal papilla of the HF, only occurs during embryonic or early postnatal development, but never in adult skin under homeostatic conditions (Millar, 2002; Sennett and Rendl, 2012). Only in rabbits and mice with large injuries HF neogenesis has been described in adult tissues (Breedis, 1954; Billingham and Russell, 1956; Ito et al., 2007).

Once the intact skin barrier is damaged, an interplay of complex cellular and molecular mechanisms is set in place to quickly and efficiently reinstate epidermal integrity in order to reestablish the skin’s impeccable and vital barrier function. To that end, a myriad of different cell types including thrombocytes, cells of the innate and adaptive immunity, epidermal stem cells, keratinocytes, endothelial cells, neurons and, most importantly, dermal fibroblasts synergistically partake in this intricate phenomenon of wound healing (Gonzalez et al., 2016; Correa-Gallegos and Rinkevich, 2022). Most importantly, mesenchymal responses are indispensable for tissue regeneration (Grose and Werner, 2003; Jiang and Rinkevich, 2020).

## 2 Contribution of distinct fibroblast subsets to skin physiology and wound healing

Mouse skin dermis comprises at least two functionally distinct fibroblast lineages which develop from a common progenitor: the papillary fibroblasts which contribute to the upper dermis including hair-follicle-associated fibroblasts of the dermal sheath (DS) and dermal papilla (DP) and are essential for HF development, and the reticular fibroblasts giving rise to adipocytes and preadipocytes of the hypodermis (Driskell et al., 2013). *In vivo* lineage tracing revealed that fibroblast diversification toward functional lineages occurs before embryonic day 16.5 (E16.5) (Driskell et al., 2013), possibly as early as E12.5 (Jacob et al., 2023). Additional heterogeneity within these two fibroblast lineages was demonstrated by scRNA-Seq of adult mouse skin (Joost et al., 2020). Recent spatial and single cell transcriptomic data confirm that also human skin harbors two major fibroblast subsets (Philippeos et al., 2018; Tabib et al., 2018; Korosec et al., 2019; Vorstandlechner et al., 2020). While differences in fibroblast subsets and markers exist between mouse and human skin, the two main subsets are functionally similar, e.g. reticular fibroblasts can undergo adipogenic differentiation, while papillary cannot (Driskell et al., 2013; Korosec et al., 2019). Reticular fibroblasts represent the majority of fibroblasts in adult tissue (Driskell et al., 2013; Lichtenberger et al., 2016) and have been shown to play a predominant role in fibrosis and cutaneous wound healing (Driskell et al., 2013; Rinkevich et al., 2015; Mastrogiannaki et al., 2016; Jiang and Rinkevich, 2020).

In mammalian postnatal skin, tissue damage that exceeds the epidermis and reaches into the papillary dermis, the upper layer of the skin dermis, or beyond, usually results in healing by scarring, which entails the reestablishment of the skin’s basic barrier to external threats and water loss while failing to restore its original architecture (Correa-Gallegos and Rinkevich, 2022). Scarred skin lacks secondary appendages such as HFs and sebaceous glands but also tensile strength and the skin’s original resilience to injury, as a dense plug of often stiff scar tissue is produced to seal the wound immediately (Erickson and Echeverri, 2018). During wound closure, resident dermal fibroblasts strongly proliferate and invade the wound edges. Using adhesive wound bed fibronectin as a scaffold for migration, these fibroblasts manage to perambulate the coagulation tissue to reach the wound bed (Yates et al., 2012), where they produce collagens and other extracellular matrix (ECM) proteins such as hyaluronic acid, fibronectin and tenascin C, thus, ultimately forming the bulk of connective tissue that constitutes the granulation tissue of the emerging scar (Sorg et al., 2017). Importantly, Driskell et al. demonstrated that this initial phase of wound repair and ECM deposition is primarily mediated by Dlk1^+^ fibroblasts of reticular origin, while Blimp1^+^ fibroblasts of papillary origin repopulate the wound bed only when a neo-epidermis has already formed (Driskell et al., 2013). In line, Rinkevich et al. showed that fibroblasts with transient Engrailed-1 expression during embryogenesis (En1 lineage positive fibroblasts, EPFs), represent the matrix-producing fibroblast lineage that mediates the deposition of granulation tissue and that likely corresponds to the reticular lineage (Rinkevich et al., 2015). Recent scRNA-Seq analyses delineated multiple unique wound fibroblast populations in regenerating murine wounds, including a rare fibroblast subpopulation that derives from myeloid progenitor cells and that gives rise to specific regenerated wound adipocytes (Guerrero-Juarez et al., 2019). The release of TGFβ by immune cells induces a transition from reticular fibroblasts into activated fibroblasts, so called “contractile myofibroblasts” (Bochaton-Piallat et al., 2016). These activated fibroblasts together with their ECM are responsible for wound contraction and new collagen and ECM deposition, which will subsequently form the scar. Apart from TGFβ signaling, the Wnt/β-catenin pathway has been shown to be a key player in dermal wound repair. While Wnt/β-catenin activity plays a critical role in epidermal stem cell maintenance, HF morphogenesis and regeneration (Andl et al., 2002; Fuchs and Raghavan, 2002; Clevers, 2006; Klaus and Birchmeier, 2008; Holland et al., 2013; Lien and Fuchs, 2014; Gonzales and Fuchs, 2017), it is also well known to have opposite effects in fibroblasts, where Wnt-signaling mediates skin fibrosis and pathological scarring (Sato, 2006; Akhmetshina et al., 2012; Hamburg-Shields et al., 2015; Lichtenberger et al., 2016; Mastrogiannaki et al., 2016).

**FIGURE 1:**
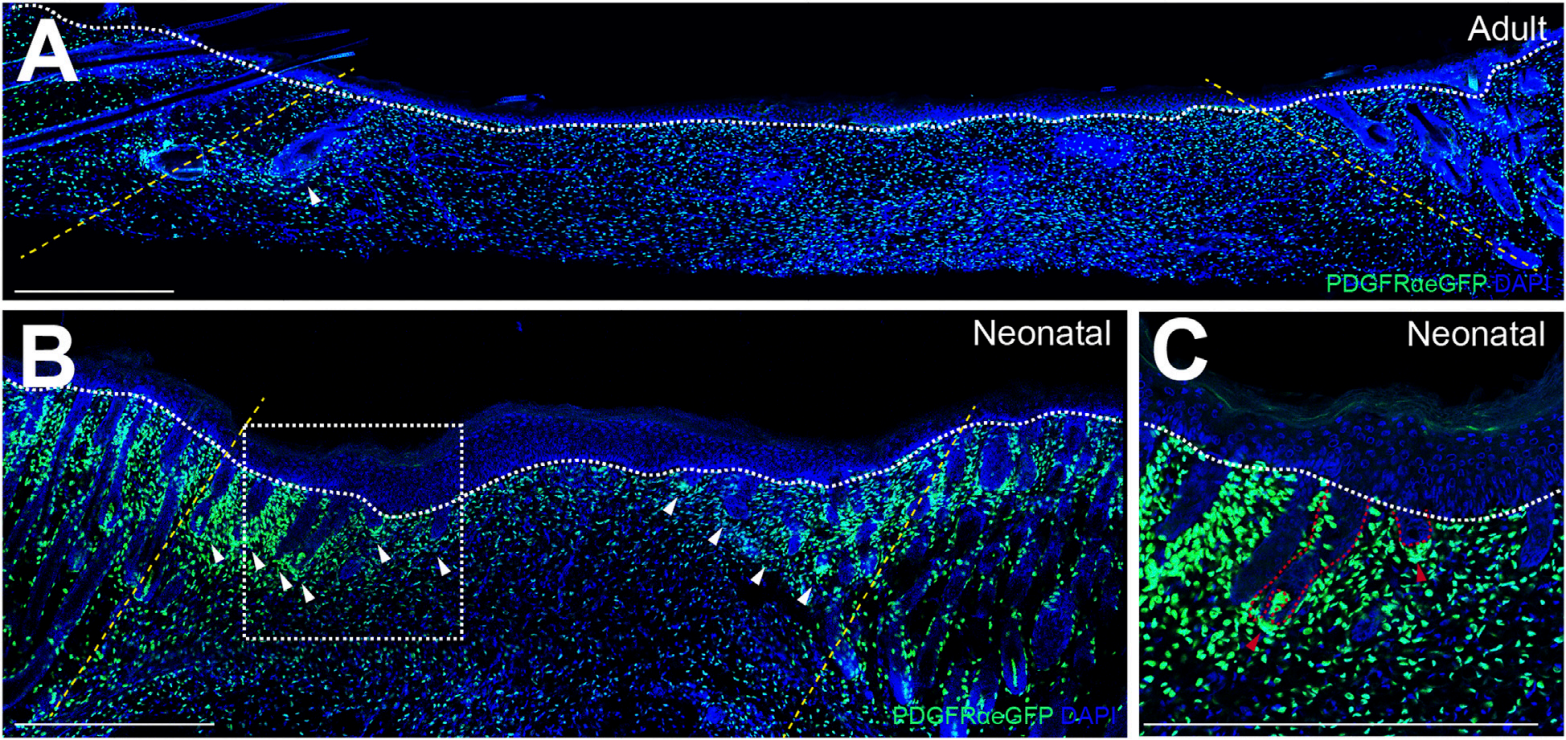
Wound-induced hair follicle *de novo* formation is more frequent in neonatal than in adult wound beds. Immunofluorescence-stainings of reepithelialized wound beds from **(A)** adult wounds 14 days after injury, **(B)** neonatal wounds 7 days after injury and **(C)** magnification of *de novo* hair follicles (HFs) in neonatal wound beds. **(A)** and **(B)** Yellow dotted lines indicate wound margins, white dotted lines demarcate the border between epidermis and dermis, and white arrows indicate *de novo* HFs. **(C)** Red dotted lines demarcate epithelial and mesenchymal compartments of *de novo* HFs and red arrows indicate *de novo* dermal papillae. Scale bars represent 500 µm.

The wound-induced hair follicle neogenesis (WIHN) model provides an outstanding platform for exploring mammalian skin regeneration. This concept was first introduced in 1956, when Billingham et al. observed HF neogenesis at the center of wounds in rabbits that were too large to close entirely by contraction (Billingham and Russell, 1956). WIHN closely recapitulates the molecular and morphological events of embryonic HF development, resulting in functional HFs comprising all cell types present in normal HFs. Of note, WIHN is much more frequent in neonatal wounds than in injuries of adult skin (Rognoni et al., 2016), see Figure 1. Like HF neogenesis during skin development, also WIHN depends on tightly regulated reciprocal signaling between epidermal cells and fibroblasts of the underlying dermis.

## 3 Embryonic signaling pathways are activated in WIHN

Importantly, certain non-mammalian species such as amphibians or flatworms manage to regenerate entire limbs or even their full body, respectively, after injury (Reddien, 2018; Dwaraka and Voss, 2021). Scarless healing in mammals, however, is a much more infrequent phenomenon and only fetal and early neonatal wounds exhibit an inherent capacity to heal by complete regeneration (Leavitt et al., 2016). The Wnt/β-catenin signaling pathway, as one of the main embryonically activated pathways during skin morphogenesis, has become one of the main protagonists for the reconstitution of a fetal- or neonatal-like regenerative skin state. For instance, Collins et al. demonstrated that a neonatal-like state of the skin dermis with prominent fibroblasts proliferation, a production of a neonatal-like extracellular matrix and the formation of ectopic HFs can be induced by epidermal activation of β-catenin (Collins et al., 2011). Importantly, this *de novo* HF formation from sebaceous gland and interfollicular epithelial cells upon transient epidermal β-catenin activation depends on the Hedgehog (Hh)-signaling pathway in unwounded skin (Silva-Vargas et al., 2005).

Correspondingly, epidermal Wnt-signaling has prominently been implicated in WIHN as well. First, Ito et al. demonstrated that epidermal overexpression of Wnt7a increased WIHN at the centre of large murine adult wounds (Ito et al., 2007). Several more recent reports support these data. For instance, Wang et al. demonstrated that tumor-necrosis factor alpha (TNFα) produced by wound-infiltrating macrophages promotes the activation of β-catenin via AKT and subsequently entails the expansion of Lgr5^+^ HF stem cells and the *de novo* formation of HFs in healing wound beds (Wang et al., 2017). Furthermore, inhibition of CXXC5, which is overexpressed in HFs of human balding skin and acts as a prominent antagonist to Wnt-signaling, led to hair regrowth and increased WIHN (Lee et al., 2017).

The modulation of other embryonic signaling pathways such as the Hh-signaling pathway has become of equal interest when it comes to the restoration of a neonatal-like skin state. Importantly, Sun et al. demonstrated that Hh-activation in naturally hairless paw skin led to the formation of neogenic HFs, however only upon concomitant activation in both, the epidermis and the dermis. Epidermal or dermal activation alone produced HF-associated tumors or the formation of a stromal condensate without subsequent HF formation, respectively (Sun et al., 2020). A recent report has established the muscle segment homeobox or Msx gene family as an important contributor to WIHN, as epidermal deletion of Msx inhibited HF *de novo* formation in large adult wounds. Importantly, in contrast to its prominent role in embryonic HF morphogenesis, modulation of bone-morphogenic protein (BMP)-signaling did not affect WIHN, which suggests that wound bed regeneration is independent of BMP-signaling (Hughes et al., 2018).

As previously described, inflammatory cells in the wound bed such as macrophages (Gay et al., 2020) and released factors like CXXC5 (Lee et al., 2017) strongly interfere in wound bed regeneration. In line, IL1β derived from wound-bed colonizing bacteria such as *Staphylococcus aureus* was shown to promote WIHN (Wang et al., 2021). Furthermore, dsRNA that is released into the wound microenvironment from damaged cells binds to TLR3, which entails subsequent activation of canonical embryonic signaling pathways such as the EDAR-, Hh- and Wnt-signaling pathways and increased WIHN (Nelson et al., 2015). Non-coding dsRNA moreover positively influences wound bed regeneration and *de novo* HF formation by upregulating retinoic-acid synthesis via TL3-activation (Kim et al., 2019) as well as through promotion of Wnt7b expression mediated by upregulation of wound bed prostaglandins (Zhu et al., 2017). Moreover, IL36α has been implicated in WIHN as its expression spikes surrounding neogenic HFs and treatment with recombinant IL36α promoted WIHN via the IL6/Stat3 pathway (Gong et al., 2020).

## 4 Manipulating embryonic signaling pathways in fibroblasts to restore regeneration

Various studies have addressed the role of Wnt-signaling in fibroblasts in the context of wound regeneration. Intriguingly, Rognoni et al. showed that the postnatal loss of hair forming ability correlated with a significant upregulation of Wnt-signaling in healing dermis (Rognoni et al., 2016). Inhibition of β-catenin-signaling in dermal fibroblasts of adult mice led to increased HF *de novo* formation in the wound, whereas β-catenin activation reduced HF regeneration in neonatal wounds (Rognoni et al., 2016; Lim et al., 2018), suggesting that highly activated dermal Wnt-signaling during wound closure might be responsible for scarce WIHN in adult skin. Correspondingly, macrophage-mediated phagocytosis of the Wnt-inhibitor SFRP4 promoted a non-regeneratory fibrotic wound phenotype while abrogation of SFRP4-phagocytosis promoted regeneration and WIHN by reducing dermal Wnt-signaling (Gay et al., 2020). In contrast to these reports, Phan et al. observed enhanced skin repair and increased WIHN upon Lef1 overexpression and Wnt-activation in Twist2-expressing dermal fibroblasts (Phan et al., 2020). Along the same line, Gay et al. reported that γδ T cell-derived FGF9 triggered Lef1 expression in dermal fibroblasts, and enhanced WIHN (Gay et al., 2013). Mascharak et al. reported that disrupting YAP-dependent mechanotransduction induces regenerative repair by fibroblasts with activated Trps1 and Wnt signaling (Mascharak et al., 2022). These contradicting results might be explained by different Lef/Tcf co-transcription factors that direct a context-dependent Wnt/β-catenin-specific response. Thus, the role of dermal Wnt-signaling in WIHN and regeneration during wounding needs to be explored in more detail.

Another important embryonic signaling pathway is the Hh pathway. Intriguingly, modulation of epidermal or dermal Hh-signaling polarized dermal cells towards a dermal papilla fate and thereby promoted a regenerative wound phenotype with prominent WIHN (Lim et al., 2018). Frech et al. were able to observe a dual role for dermal Hh-signaling in distinct dermal fibroblasts lineages during wound healing. In fact, they demonstrated that Hh-signaling in the reticular fibroblast lineage promotes wound closure, possibly via increased angiogenesis and the expansion of PDGFRα^+^ wound bed fibroblasts, while Hh-signaling in the papillary fibroblast lineage is essential for the expression of HF morphogenesis-associated genes and *de novo* HF formation within the healing wound (Frech et al., 2022). Accordingly, scRNA-Seq revealed that direct effectors of Hh-signaling, such as Gli1, are solely active in fibroblasts within the central area of large wounds, where HF neogenesis occurs (Abbasi et al., 2020). This study also suggested that retinoic acid (RA) and Runx1 are master regulators of WIHN, and demonstrated that fibroblast-specific deletion of the quiescence-associated factor hypermethylated in cancer 1 (Hic1) enhanced WIHN by increasing fibroblast density in the early repair phase of large wounds. Altogether, these data indicate that Hh-signaling contributes to the mesenchymal competence for *de novo* HF regeneration within interfollicular epidermis, and that many other factors with regeneration-enhancing capability remain to be identified for future therapeutic approaches.

## 5 Papillary fibroblasts at the bifurcation of regeneration and scarring

**FIGURE 2:**
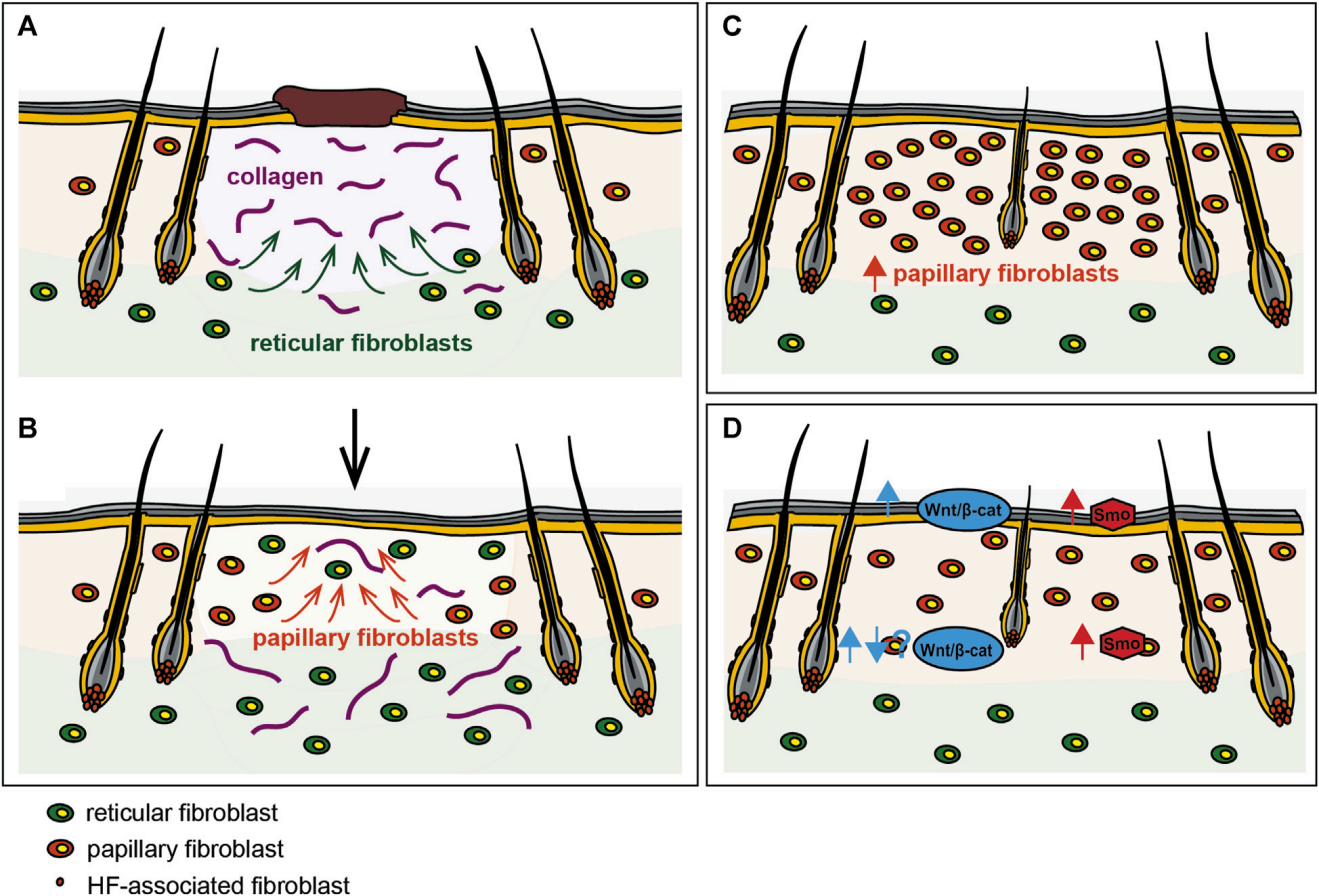
Reinstating a neonatal-like state during wounding promotes regeneration. **(A)** The reticular fibroblast lineage mediates the initial phase of wound repair including the deposition of collagens and other components of the ECM, while **(B)** the papillary fibroblast lineage repopulates the wound bed only at later stages of wound repair. Manipulation of the dermal cellular composition like **(C)** expanding the papillary fibroblast lineage or **(D)** modulation of embryonic signaling pathways such as the Wnt- and Hh-signaling pathways in fibroblasts increases WIHN and regeneration during wounding.

The papillary fibroblast lineage not only gives rise to all HF-associated mesenchymal cells such as the dermal papilla and the dermal sheath, it is also essential for HF development during embryogenesis (Driskell et al., 2013; Gupta et al., 2019; Mok et al., 2019). Papillary and reticular fibroblasts are not only functionally distinct, they also respond to different epidermis-derived signals in a unique manner (Lichtenberger et al., 2016). As WIHN recapitulates the major steps of embryonic HF morphogenesis, it is therefore not surprising that the papillary fibroblast lineage has proven crucial in this context as well. While it has long been postulated that HFs might be a source of mesenchymal cells with regenerative potential during wound healing (Jahoda and Reynolds, 2001), and a subset of HF-associated fibroblasts continuously replenishes HFs with fibroblasts as they undergo repeated cycles of degeneration, remodeling, and regeneration (Rahmani et al., 2014), these HF-associated fibroblasts migrate into the wounds but only contribute a minority of cells to newly formed HFs and the granulation tissue (Abbasi et al., 2020). Instead, fibroblasts of the upper interfollicular dermis (fate-mapped with the quiescence-associated factor hypermethylated in cancer 1, Hic1) contribute largely to both the mesenchymal compartment of neogenic HFs and the wound neo-dermis (Abbasi et al., 2020). In their study, Rognoni et al. demonstrated that impaired hair forming ability correlated with the age-dependent reduction in the abundance of papillary fibroblasts (Rognoni et al., 2016). Accordingly, single-cell transcriptomics revealed that WIHN depends on upper dermal fibroblasts that are closely related to cells of the DP at transcriptomic level, and that migrate from the wound edge towards the wound centre, where WIHN typically occurs (Phan et al., 2021), thus indicating that increasing papillary fibroblast density within the wound might promote WIHN (see Figure 2C). In fact, increasing the number of papillary fibroblasts in adult skin to neonatal levels prior to wounding via transient Wnt/β-catenin activation in the epidermis, restored the skin’s ability to form new HFs within the wound (Driskell et al., 2013). Rinkevich et al. discovered that during embryogenesis En1 lineage negative fibroblasts (ENFs), which are the dominant population in the developing dermis, minimally contribute to scar formation in adult wounds (Rinkevich et al., 2015). Transplantation of purified ENFs into donor wounds, led to a phenotypic transition of wounds from scarring to regeneration (Jiang et al., 2018). Importantly, Hh-signaling in the papillary fibroblast lineage but not the reticular fibroblast lineage is crucial for HF regeneration during wound healing (Frech et al., 2022). While it has been suggested that reprogramming scarring fibroblasts during wound healing might contribute to a more regenerative wound phenotype that entails WIHN (Mascharak et al., 2022), the majority of data points towards the expansion of fibroblasts of papillary origin itself, either via activation of embryonic signaling pathways and subsequent proliferation (Lichtenberger et al., 2016; Rognoni et al., 2016; Phan et al., 2020) or via transplantation (Rinkevich et al., 2015), or by inhibiting reticular fibroblasts, as the key event that mediates the switch from scarring to regenerative healing within the wound bed.

## 6 Summary and Perspectives

Regeneration is the best possible outcome of tissue repair, but skin injury typically leads to fibrotic, non-functional scars. Our inability to regenerate fully functional skin and the appendages contained within the dermis is a major impediment to human skin wound healing. Apart from imperfectly healed wounds in adult skin, the aging-related decline in the skin’s capability to heal completely and skin fragility are a major risk of morbidity in the elderly. Similar to mouse skin, also in human skin the number of fibroblasts, specifically that of papillary fibroblasts, declines as we age, concomitant with accumulating senescent fibroblasts, resulting in skin thinning (Harper and Grove, 1979; Gilchrest, 1982; Gilchrest, 1996; Varani et al., 2001) and a progressive loss of the adipocyte layer (Kruglikov and Scherer, 2016). Importantly, aging not only affects the expression of a plethora of genes in fibroblasts (Haydont et al., 2019a; Haydont et al., 2019b; Sole-Boldo et al., 2020) but also substantially reduces the interactions of dermal fibroblasts with other skin cell types (Sole-Boldo et al., 2020), which likely further affects skin function. Since WIHN is age-dependent, reactivation of embryonic signals in aged fibroblasts might also counteract impaired wound healing. Alopecia and chronic wounds are also debilitating complications of diabetes mellitus (Liu et al., 2022). Intriguingly, like hair growth and cycling, also WIHN was affected in diabetic mice but could be restored by a small molecule activating Wnt/β-catenin signaling (Ryu et al., 2022). Interestingly, recent findings suggest that high glucose levels also affect the immunomodulatory functions of fibroblasts (Al-Rikabi et al., 2021; Liu et al., 2022). The development of pro-regenerative therapies requires detailed understanding of the cellular and molecular events that determine if an injury heals with fibrosis and scarring, or regenerates scarlessly towards a fully functional skin comprising its appendages. Since the overall architecture of murine and human skin are similar and the major fibroblast subsets in both tissues are alike, the WIHN model is an excellent tool to study how tissues mobilize and coordinate distinct cell populations to initiate embryonic-like skin regeneration. This model has already provided important insights into the cellular and molecular mechanisms of wound healing, and thus for the development of future regenerative medicine approaches. Both, increasing papillary fibroblast density and modulating dermal signaling seem to be promising approaches to shift wound repair with scarring towards complete regeneration. Reactivating high-fidelity tissue morphogenesis signaling pathways in adults following a severe injury of the skin could replace or reduce the need for transplantation therapies.
